# Partial Purification and Characterization of Bacteriocin-Like Inhibitory Substances Produced by *Streptomyces* sp. Isolated from the Gut of *Chanos chanos*

**DOI:** 10.1155/2021/7190152

**Published:** 2021-12-14

**Authors:** Muhammad A. Kurnianto, Harsi D. Kusumaningrum, Hanifah N. Lioe, Ekowati Chasanah

**Affiliations:** ^1^Food Science Study Program, Graduate School, IPB University, Bogor, Indonesia; ^2^Department of Food Technology, Faculty of Engineering, Universitas Pembangunan Nasional “Veteran” Jawa Timur, Surabaya, Indonesia; ^3^Department of Food Science and Technology, Faculty of Agricultural Engineering and Technology, IPB University, Bogor, Indonesia; ^4^Research and Development Center for Marine and Fisheries Product Processing and Biotechnology, Ministry of Marine and Fisheries, Jakarta, Indonesia

## Abstract

Bacteriocin-like inhibitory substances (BLIS) have sparked great interest because of their promising use in food as natural antimicrobial agents. In this work, six *Streptomyces* isolates obtained from the gut of *Chanos chanos* demonstrated their ability to produce extracellular metabolites with inhibitory activity against *Salmonella enterica* serovar Typhimurium, *Escherichia coli*, *Listeria monocytogenes*, and *Staphylococcus aureus*. Exposure of the extracellular metabolites to proteolytic enzymes (i.e., proteinase-K, trypsin, and pepsin) revealed high sensitivity and confirmed their proteinaceous nature. The metabolites were stable at high temperatures (up to 100°C for 30 min) and a wide range of pH (pH 2.0–7.0). Fractionation of the crude BLIS by filtration yielded three fractions based on molecular weight: <3 kDa, 3–10 kDa, and >10 kDa. Analysis of the antibacterial activity of these fractions showed increased specific activity, especially in the fraction with a molecular weight (MW) of <3 kDa, relative to the crude sample. The fraction with MW < 3 kDa had minimum inhibitory and bactericidal concentrations in ranges 0.04–0.62 mg·mL^−1^ and 0.08–1.25 mg·mL^−1^, respectively. This fraction also showed better temperature and pH stability compared with crude BLIS. Brine shrimp toxicity assay revealed that this fraction has moderate toxicity with a 50% lethal concentration of 226.975 *μ*g·mL^−1^ (i.e., moderate toxicity) to *Artemia salina*. Identification of the peptide sequences of this fraction by liquid chromatography–tandem mass spectrometry yielded 130 proteins with retention times of 15.21–19.57 min. Eleven proteins with MWs of 1345.66–2908.35 Da and composed of less than 30 amino acid residues with high hydrophobicity (15.34–26.22 kcal·mol^−1^) appeared to be responsible for the antibacterial activity of the fraction. This study revealed the potential application of BLIS from *Streptomyces*, especially BLIS SCA-8, as antibacterial agents.

## 1. Introduction

Increasing consumer awareness of the adverse health effects of chemical preservative in food has resulted in heightened interest in fresh and minimally processed food [1–3]. However, this trend presents a massive challenge because minimally processed food is closely related to various diseases caused by pathogenic bacteria [4, 5]. Hence, numerous explorations of potential natural preservatives, such as bacteriocins or bacteriocin-like inhibitory substances (BLIS), have been conducted in efforts to replace chemical preservatives to improve the shelf life and safety of food [6, 7].

Bacteriocins are short-chain protein or polypeptide compounds synthesized ribosomally by microorganisms that could inhibit the growth of bacterial strains closely related to the bacteriocin-producing strains [7, 8]. The term BLIS describes a peptide or protein antimicrobial compound that is synthesized by ribosomes but not fully characterized by its amino acid sequence and biochemical properties [9]. Bacteriocins may be divided on the basis of their structure into three main classes: class I includes lantibiotics, class II includes small posttranslationally unmodified bacteriocins, and class III includes large antibacterial proteins (>10 kDa) and bacteriolysins [10]. Bacteriocins generally have a narrow antibacterial spectrum and are limited to closely related genera or species [7]. However, the growing demand for natural agents for food preservation (i.e., to replace chemical preservatives) or clinical applications renders the study and exploration of bacteriocins with broad spectrum antibacterial activity increasingly necessary [11, 12].

Most bacteriocins are produced by lactic acid bacteria, such as those belonging to *Lactococcus*, *Lactobacillus*, *Streptococcus*, and *Pediococcus* [7, 10]. Some bacteria from other genera, one of which is *Streptomyces*, are also capable of producing bacteriocins or BLIS with potential antibacterial activity and stability [13]. Several authors have examined the bacteriocins produced by *Streptomyces pluripotens* sp. nov. [14], *Streptomyces scopuliridis* sp. nov. [15], *Streptomyces griseus* (grisemycin) [16], and *Streptomyces bottropensis* (bottromycin A2) [17]. These studies show that *Streptomyces* bacteriocins have broad spectrum antibacterial activity against Gram-positive and negative bacteria and even resistant pathogenic bacteria, such as methicillin-resistant *Staphylococcus aureus* [14]. However, although the potential activity of some BLIS produced by *Streptomyces* has been reported, many other *Streptomyces* species producing these substances have not been described [13].

*Streptomyces* is a Gram-positive bacterium widely known to produce various bioactive compounds [18]. These bacteria are widely abundant in various ecosystems and can form associations with eukaryotic hosts, such as fish [19, 20]. This ability confers the bacterium with the physiological and genetic ability to adapt and express metabolites of varying bioactivity [21]. Several studies have discovered metabolites with potential bioactivity produced by *Streptomyces* associated with fish [20, 22, 23]. Kurnianto et al. [23, 24], for instance, identified a suspected peptide-containing antibacterial metabolite with broad spectrum antibacterial activity produced by *Streptomyces* associated with *Chanos chanos*.

The present study is aimed at analyzing the potential of six *Streptomyces* isolates from the gut of *C. chanos* to produce BLIS, at analyzing the stability and antibacterial activity characteristics of BLIS, and at identifying the active fraction of BLIS using liquid chromatography-tandem mass spectrometry (LC-MS/MS).

## 2. Materials and Methods

### 2.1. Bacterial Strains and Culture Collection

Six *Streptomyces* (i.e., *S. variabilis* SCA-5, *S. variabilis* SCA-11, *S. variabilis* AIA-10, *S. labedae* SCA-8, *S. globisporus* AIA-12, and *S. misionensis* AIA-17) were obtained from the gut of milkfish (*C. chanos*) [23, 24]. The strains were identified through their morphological, physiological, biochemical, and molecular characteristics by 16S rRNA gene sequencing. The isolates were cultivated in ISP-2 medium (yeast extract, 4 g; malt extract, 10 g; dextrose broth, 4 g; bacteriological agar, 20 g; and distilled water, 1,000 mL) and incubated at 30°C. Target bacteria, including *Salmonella enterica* serovar Typhimurium ATCC 14028, *Escherichia coli* ATCC 25922, *Listeria monocytogenes* ATCC 35152, and *S. aureus* ATCC 25923, were grown on tryptic soy broth (Oxoid, UK) and incubated for 24 hours at 37°C.

### 2.2. Production and Preliminary Screening of BLIS-Producing Bacteria

The six *Streptomyces* isolates were inoculated on ISP-2 and incubated at 30°C during the optimum antibacterial production time (9–11 days) with agitation in a shaker incubator (New Brunswick, Germany) [24]. Bacterial cultures were centrifuged (Hermle, Germany) at 7,000 × *g* and 4°C for 15 min to separate the supernatant from the cell biomass. The supernatants were adjusted to pH 6.5–7.0 with 1 mol·L^−1^ NaOH (Merck, Germany) and passed through a 0.22 *μ*m membrane filter (Sartorius, France) to obtain a neutralized cell-free supernatant (CFS). This CFS was analyzed for its inhibitory activity against two bacteria (i.e., *E. coli* ATCC 25922 and *S. aureus* ATCC 25922) via the microdilution method [25]. A total of 100 *μ*L of the tested bacterial suspension (1 × 10^6^ CFU · mL^−1^), 80 *μ*L of Mueller–Hinton broth medium (MHB) (Oxoid, UK), and 20 *μ*L of the CFS were added to each well of a microplate. Test bacteria grown on MHB medium without exposure to the CFS were used as controls. The microplate was incubated for 24 h at 37°C, and the absorbance of each well of the microplate was measured at 600 nm using an ELISA reader (Bio-Rad, US). The growth inhibitory activity of the test bacteria was calculated from the absorbance of samples with and without treatment (control). An absorbance value lower than the control indicates inhibition of the growth of the test bacteria. CFS samples showing potential activity were concentrated using a freeze dryer (Christ, Germany) to obtain crude BLIS.

### 2.3. Confirmation of the Proteinaceous Nature of Crude BLIS

The proteinaceous nature of crude BLIS was assessed by exposure of the six concentrated CFS samples to various enzymes, including proteinase-K (Geneaid, Taiwan), trypsin (Sigma-Aldrich, USA), and pepsin (Sigma-Aldrich), at a final concentration of 10 mg·mL^−1^ and incubation for 2 h at 37°C. The samples were heated for 2 min to inactivate the enzyme, cooled to room temperature, and then subjected to antimicrobial activity testing [11] by using the agar well diffusion method.

### 2.4. Partial Purification via Ultrafiltration Membranes

The six crude BLIS samples were separated according to molecular weight by using 3 and 10 kDa ultracentrifuges (Amicon Ultra Centrifugal Unit, Regenerated Cellulose 3,000 and 10,000 MWCO, Merck Millipore Ltd., Germany) for 60 min at 1,328.4 × *g* and 4°C. This process separated the samples into three BLIS ultrafiltration fractions (BLIS-UF), i.e., <3 kDa, 3–10 kDa, and >10 kDa [26]. Each fraction was concentrated with a freeze dryer (Christ, Germany), adjusted to a final volume of 80 mL with deionized water, and then subjected to bacterial inhibitory activity tests to assess their bioactivity.

### 2.5. Quantification of Total Protein

The total protein concentration of each sample was determined on the basis of the Bradford method (Bradford 1976), which refers to Jamaluddin et al. [27]. A total of 160 *μ*L of each sample was reacted with 40 *μ*L of Bradford reagent in a 96-well microtiter plate and then incubated at 37°C in an orbital shaker for 10 min. Protein concentrations were determined by an ELISA reader (Bio-Rad) at 595 nm. Distilled water was used as a blank sample, and a protein standard curve was obtained using bovine serum albumin as a protein standard.

### 2.6. Determination of the Antibacterial Activity of BLIS

The antibacterial activity of BLIS was determined using the agar well diffusion method. A total of 25 mL of Mueller–Hinton agar (Oxoid, UK) containing the test bacteria (1% *v*/*v*; *E. coli*, *S.* Typhimurium, *L. monocytogenes*, and *S. aureus*) at a density of 1 × 10^6^ CFU · mL^−1^ was poured into the wells of a plate and allowed to solidify. A well (diameter, 6 mm) was made in the agar with a cork borer, and 100 *μ*L samples of BLIS were placed into the wells. After incubation at 37°C for 24 h, the diameter of the inhibition zone (mm) was measured. Distilled water and ampicillin 10 *μ*g·mL^−1^ were used as controls. The antibacterial activity of BLIS was expressed as activity (*A*) and specific activity (*B*) [28]. Purification fold (*C*) and yield activity (%) (*D*) of the samples were also calculated.

BLIS activity (*A*) was calculated as shown in equation (1) and expressed in units of AU·mL^−1^, where 1 AU·mL^−1^ was defined as the unit area of the inhibition zone per unit volume (mm^2^·mL^−1^). In equation (1), *L*_*z*_ refers to the clear zone area (mm^2^), *L*_*s*_ refers to the well area (mm^2^), and *V* refers to the sample volume (mL).

(1) $$\begin{array}{c} A=\frac{L_{z}-L_{s}}{V}. \end{array}$$

BLIS-specific activity (*B*) was defined as the ratio of the total BLIS activity of the sample to the total protein concentration (AU·*μ*g^−1^) and calculated using equation (2), where *A* refers to the BLIS activity (AU·mL^−1^), *T*_*p*_ refers to the total protein concentration (*μ*g) in the sample, and *V* refers to the sample volume (mL).

(2) $$\begin{array}{c} B=\frac{A\times V}{T_{p}\times V}. \end{array}$$

Purification fold (*C*) was defined as the ratio of the specific activity of the fraction obtained at each purification step (*B*_*p*_) to the specific activity of crude BLIS (*B*_0_) and calculated using equation (3), where *B*_*p*_ was the BLIS-specific activity of the fraction obtained at each steps of purification and *B*_0_ was the BLIS-specific activity of crude BLIS.

(3) $$\begin{array}{c} C=\frac{\;B_{p}}{\;B_{0}}. \end{array}$$

Yield activity (*D*) was defined as the ratio of the total BLIS activity (AU·mL^−1^) calculated at each step of purification to the total activity of crude BLIS and calculated using equation (4), where *A* was the BLIS activity (AU·mL^−1^) in each step, *V* was the sample volume (mL), and *A*_0_ was the BLIS activity of crude BLIS.

(4) $$\begin{array}{c} D=\left(\frac{\;A\times V}{\;A_{0}}\right)\times 100. \end{array}$$

### 2.7. Analysis of Minimum Inhibitory and Bactericidal Concentrations

Determination of the MIC of BLIS was conducted by preparing BLIS samples at several concentrations (i.e., 10, 5.0, 2.5, 1.25, 0.625, 0.312, 0.156, 0.078, 0.04, 0.02, 0.009, and 0.004 mg·mL^−1^). Each BLIS sample was placed on a microplate that had previously been inoculated with 0.1 mL of test bacteria (density, 1 × 10^6^) and 0.1 mL of MHB (HiMedia, India). The microplate was incubated for 24 h at 37°C. The growth of the test bacteria on the microplate was observed using a microplate reader (Bio-Rad) at 600 nm, and their viability was determined by the plate counting method [3]. The MIC was obtained from the lowest concentration of bacteriocin that could inhibit the growth of the test bacteria. The MBC of BLIS was evaluated as follows. After MIC determination, the liquid remaining on the microplate was inoculated with Mueller–Hinton agar (HiMedia) and then incubated for 24 h at 37°C. The MBC refers to the MIC of bacteriocin required to kill 99.9% of the tested bacteria.

### 2.8. Determination of the Temperature and pH Stability of BLIS

Evaluation of the temperature stability of BLIS was performed by dissolving all of the fractions in deionized water and then incubating the resulting solutions at 60, 80, or 100°C for 30 min or 121°C for 15 min. The solutions were cooled to room temperature and subjected to antibacterial activity tests [29]. The pH stability of BLIS was determined by adjusting the pH of the purified fractions to pH 2.0–10.0 by using 1 mol·L^−1^ HCl and 1 mol·L^−1^ NaOH as necessary and incubating for 2 h. Prior to the antibacterial activity test, the pH of the samples was set to pH 7.0. All antibacterial activity tests were performed by using the agar-well diffusion method, as described above.

### 2.9. Analysis of Toxicity by Using Brine Shrimp Lethality Assay

*Artemia salina* cysts were hatched in artificial seawater containing 27 g of NaCl in 3 L of distilled water and provided light and aeration. The larval hatching process was conducted for 48 h. Once hatched, the larval eggs were considered ready for analysis. The purified BLIS fraction showing the best specific activity was dissolved in artificial seawater to obtain test solutions with final BLIS concentrations of 2,000, 1,000, 200, and 20 *μ*g·mL^−1^. A total of 1 mL of each test solution was transferred to a separate test tube and added with 10 two-day-old *A. salina* larvae by using a sterile syringe. The volume in the test tube was adjusted to 2 mL to achieve final BLIS concentrations of 1 mg·mL^−1^, 500 *μ*g·mL^−1^, 100 *μ*g·mL^−1^, and 10 *μ*g·mL^−1^. The test tubes were incubated for 24 h, and the number of dead *A. salina* larvae was counted. The lethal concentration (LC_50_) of BLIS was determined via the probit analysis method with a 95% confidence interval using SPSS software. Each concentration was tested thrice, and artificial seawater was used as a negative control [30].

### 2.10. LC-MS/MS Analysis and *In Silico* Study of the BLIS Fraction with the Greatest Activity

The BLIS fraction with the greatest antibacterial activity was dissolved in 1 mL of water, centrifuged for 1 min, and then injected into a NanoLC Ultimate 3000 Tandem Q Exactive Plus Orbitrap HRMS instrument equipped with a Thermo PepMap RSLC C18 capillary column (75 *μ*m × 15 cm, 3 *μ*m, 100 Å) and a Thermo Scientific™ 164649 trap column (30 *μ*m, 5 mm). The sample was eluted with H_2_O with 0.1% formic acid (A) and acetonitrile with 0.1% formic acid (B) at a flow rate of 300 nL·min^−1^. The elution program was as follows: 2%–35% B for 30 min, 30%–90% B for 15 min, 90% B for 15 min, and 5% B for 30 min. Spectra were collected over the mass range of 200–2,000 m/z. The mass spectral results were analyzed using Proteome Discoverer 2.2 (Thermo Scientific), which uses the search engine SEQUEST HT, for database-based protein identification. The enzyme used was trypsin with a maximum miss cleavage of 2. The dynamic modifications applied were oxidation (for the amino acid methionine) and acetylation (for N-terminals). The percentages of hydrophobic and cationic amino acid residues were predicted using ProtParam (https://web.expasy.org/protparam/), and the physicochemical properties of the detected amino acid peptide sequences (i.e., sequence length, molecular weight (MW), hydrophobicity, and Boman index) were determined using PepDraw (https://www.pepdraw.com/).

### 2.11. Statistical Analysis

All experiments were performed as three independent replicates, and the results were expressed as mean ± standard deviation. Data analysis was performed using SPSS 18.0 software. One-way analysis of variance (ANOVA) and Duncan's multiple range test (95% confidence interval) were applied to detect significant differences between means.

## 3. Results and Discussion

### 3.1. Antibacterial Activity of Crude BLIS

**Figure 1:**
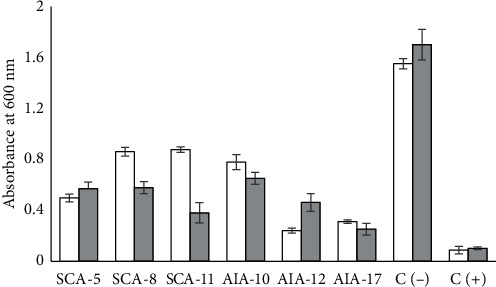
The growth absorbance (in 600 nm) of test bacteria in Mueller Hinton broth exposed to cell-free supernatant (CFS); white square: *E. coli*; light-gray square: *S. aureus*; C (−): control negative using Mueller Hinton broth inoculated with test bacteria without being exposed to CFS; C (+): control positive using ampicillin 10 *μ*g·mL^−1^.

**Figure 2:**
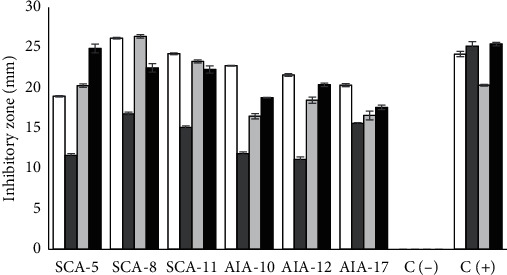
Inhibition zone (mm) of concentrated cell-free supernatant (crude BLIS) against test bacteria determined using the agar well diffusion method; white square: *E. coli*; dark-gray square: *S. aureus*; light-gray square: *L. monocytogenes*; black square: *S.* Typhimurium; C (−): control negative using distilled water; C (+): control positive using ampicillin 10 *μ*g·mL^−1^.

The neutralized CFS of six *Streptomyces* isolates showed potential inhibitory activity (Figure 1). The growth inhibition of *E. coli* and *S. aureus* was highest in the CFS of *S. globisporus* AIA12 and *S. misionensis* AIA17. Meanwhile, the lowest inhibition of growth of *E. coli* and *S. aureus* was found in CFS produced by *S. labedae* SCA-11 and *S. variabilis* AIA-10, respectively. ANOVA followed by Duncan's multiple range test indicated that the growth absorbance of test bacteria exposed to CFS was significantly lower than that of test bacteria not exposed to CFS. This result indicates that the presence of extracellular metabolites produced by *Streptomyces* in CFS could inhibit the growth of the test bacteria and supports previous findings demonstrating the potential antibacterial properties of metabolites produced by *Streptomyces* isolated from the gut of *C. chanos* [23, 24]. The difference in antibacterial activity produced is due to differences in the concentration of extracellular metabolites produced [13]. Analysis of antibacterial activity using the agar well diffusion method confirmed these findings. Specifically, the concentrated CFS formed inhibition zones against all tested bacteria (Figure 2). These results show that crude BLIS has broad spectrum antibacterial activity.

The zones of inhibition determined in this study indicated that the six crude BLIS samples have better inhibitory activity against Gram-negative bacteria than against Gram-positive bacteria. This finding contradicts most studies reporting that bacteriocins are only active against Gram-positive bacteria [31, 32]. However, recent studies have reported the ability of several bacteriocins to inhibit Gram-negative bacteria. For instance, the bacteriocins synthesized by *Streptomyces nigrescens* inhibit *Vibrio parahaemolyticus*, bacteriocin SLG10 inhibits *E. coli*, and bacteriocin JLA-9 inhibits *E. coli*, *S.* Typhimurium, *Pseudomonas fluorescens*, and *Shigella flexneri* [3, 13, 33]. According to Pei et al. [3], the inhibitory activity of bacteriocins against Gram-negative bacteria is due to the ability of these substances to form interactions with nucleic acids and bacterial intracellular enzymes and damage the integrity of bacterial cell membranes. Choi and Lee [34] added that the inhibitory activity of bacteriocins against Gram-negative bacteria may also be due to their low MW. These bacteriocins can penetrate cell membranes through porins and directly attack the intracellular components of cells.

### 3.2. Proteinaceous Nature Properties of Crude BLIS

Since bacteriocins and BLIS are considered peptides, confirmation of the proteinaceous nature of these compounds is necessary. Analysis of the proteinaceous nature of the crude BLIS revealed that the antimicrobial activity of the six extracts against *E. coli* was completely lost after treatment with proteinase-K and trypsin (Table 1). Moreover, whereas other crude BLIS fractions showed a significant decrease in activity, only the crude BLIS of *S. variabilis* SCA-11 and SCA-5 completely lost their activity following pepsin treatment. These findings are consistent with those of Lasik-Kurdyś and Sip [35], who reported that BLIS produced by *Oenococcus oeni* loses its activity after exposure to pronase E, proteinase-K, trypsin, pepsin, and *α*-chymotrypsin. Moračanin et al. [36] also found that the bacteriocin produced by *Leuconostoc mesenteroides* loses its antilisteria activity after exposure to pepsin, papain, and proteinase-K. These results confirm the proteinaceous nature of active metabolites in crude BLIS, which may be responsible for the latter's antibacterial activity.

### 3.3. Stability of Crude BLIS

**Figure 3:**
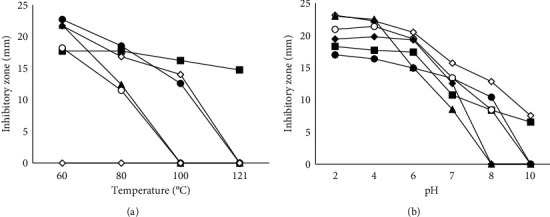
Inhibitory zone (mm) of crude BLIS after heat (a) and pH (b) treatment against *E. coli*; ∎: SCA-5; ●: SCA-8; ⬦: SCA-11; ▲: AIA-10; ○: AIA-12; ⬥: AIA-17.

Knowledge of the temperature and pH stability of BLIS is important because good stability is necessary to realize its applications in food processing. Among the crude samples assayed in this work, the crude BLIS of *S. variabilis* SCA-5 and *S. labedae* SCA-8 were the most stable (Figure 3). Both extracts could maintain over 50% of their antibacterial activity after heating up to 100°C. Even in crude BLIS of *S. variabilis* SCA-5, the activity was maintained up to 121°C. Besides, both extracts were also able to maintain their antibacterial activity at exposure to a wide range of pH 2.0–7.0. However, significant decreases in activity occurred when the temperature and pH were increased beyond these ranges. This finding is consistent with the results of Hernández-Saldaña et al. [13], who reported that the bacteriocin produced by *S. griseus* and *S. nigrescens* shows activity loss of up to 50% at 100°C and stable at pH 3–7. Meanwhile, in *S. violaceoruber* and *S. bottropensis*, the activity decreased up to 70% at the same temperature. Du et al. [31] reported that plantaricin GZ1-27 maintains its activity at temperatures of up to 80°C for 30 min and is stable at pH 2.0–6.0.

### 3.4. Antibacterial Activity of BLIS Ultrafiltered Fractions

**Figure 4:**
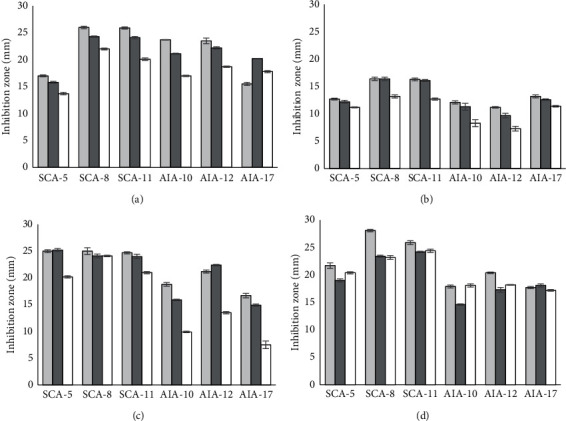
Inhibition zone (mm) of BLIS ultrafiltration fraction based on molecular weight (<3 kDa, 3–10 kDa, and >10 kDa) against *E. coli* (a), *S. aureus* (b), *L. monocytogenes* (c), and *S.* Typhimurium (d) using the agar well diffusion method; light-gray square: >10 kDa; dark-gray square: 3–10 kDa; white square: <3 kDa.

Antibacterial activity analysis using the agar well diffusion method showed that the BLIS ultrafiltered (BLIS-UF) fraction with MW > 10 kDa could produce the largest inhibition zone (Figure 4). This trend is similar to the trend of BLIS activity observed. However, BLIS-specific activity calculations indicated that this fraction has the lowest activity among the fractions obtained. BLIS-specific activity describes the ratio of BLIS activity to the total protein concentration, where this value is the activity per one *μ*g of protein contained in the fraction. The greater the BLIS-specific activity of a fraction, the more active it is [28]. Compared with the BLIS-UF fraction with MW > 10 kDa, those with MW < 3 kDa and 3–10 kDa had higher BLIS-specific activity. These results indicate that the active fraction of BLIS *Streptomyces* has a low MW, consistent with previous studies reporting that BLIS produced by *Streptomyces* have MW < 3 kDa, such as *Streptomyces griseus* IFO 1330 (1,833 kDa), *Streptomyces griseus* XylebKG-1 ADFC02 (~2 kDa), and *Streptomyces nigrescens* ATCC 23941 (3 kDa). BLIS shows inhibitory activity against *Bacillus cereus*, *Vibrio parahaemolyticus*, *Enterococcus casseliflavus*, *L. monocytogenes*, and *Micrococcus luteus* [13, 16].

Among the fractions obtained, BLIS-UF SCA-8 with MW < 3 kDa showed the highest BLIS-specific activity. This fraction revealed broad spectrum antibacterial effects against *E. coli*, *S. aureus*, *L. monocytogenes*, and *S.* Typhimurium with specific activities of 635.6, 167.9, 664.5, and 615.5 AU·*μ*g^−1^, respectively (Table 2). These results reflect an increase in BLIS-specific activity of up to 396.9 AU·*μ*g^−1^ compared with that of crude BLIS. Such results may be attributed to the purification level of crude BLIS, which was increased by up to 2.5 times. Application of ultrafiltration membranes appeared to be quite effective in separating crude BLIS into purified fractions based on MW. Yield recoveries ranged from 15.7% to 99.6%. Among the fractions obtained, that with MW < 3 kDa revealed the lowest yield. The results of the present study are similar to those of Zacharof et al. [37], who found a decrease in yield recovery from 68% to 36% after filtration using 1 kDa MWCO nanofilters.

## 4. MIC and MBC of BLIS Ultrafiltration SCA-8 with MW < 3 kDa Fraction

The MIC and MBC of BLIS-UF SCA-8 with MW < 3 kDa are shown in Table 3. This fraction revealed MICs ranging from 0.04 mg·mL^−1^ to 0.62 mg·mL^−1^ and MBCs ranging from 0.08 mg·mL^−1^ to 1.25 mg·mL^−1^. The lowest MIC of this fraction (0.04 mg·mL^−1^) was obtained in *S. aureus* and *L. monocytogenes*, and its lowest MBC (0.08 mg·mL^−1^) was observed in *S. aureus*. The MIC and MBC obtained in this study are higher than those of the bacteriocins SLG10 and JLA-9. The bacteriocin SLG10 produced by *Lactobacillus plantarum* shows MICs in the range of 0.016–0.032 mg·mL^−1^ and MBCs in the range of 0.016–0.064 mg·mL^−1^ and is most lethal to *L. monocytogenes* CICC 21529 [3]. Bacteriocin JLA-9 has MBCs in the range of 0.016–0.032 mg·mL^−1^ [33]. Differences in MICs and MBCs obtained in this study relative to those in previous reports may be related to the purity of the bacteriocins. Specifically, bacteriocins SLG10 and JLA-9 were tested in their pure form, whereas BLIS-UF SCA-8 with MW < 3 kDa is only semipurified at best. Sharma et al. [38] indicated that further purification may reduce MIC, which has implications for smaller MIC doses in certain microorganisms.

### 4.1. Stability of BLIS Ultrafiltration SCA-8 with MW < 3 kDa Fraction

Among the fractions obtained, BLIS-UF SCA-8 with MW < 3 kDa revealed the greatest BLIS-specific activity. Stability analysis of this fraction showed better stability at high temperatures and pH, as well as improved sensitivity to enzymes, compared with crude BLIS. BLIS-UF SCA-8 with MW < 3 kDa retained over 70% of its antibacterial activity despite exposure to temperatures of 60–100°C for 30 min and solution pH of 2.0–7.0. It also showed enhanced sensitivity to pepsin (Table 4). Considering that food materials comprise various matrices and are subjected to various processing methods, such as heating or acidification [39, 40], the excellent stability of BLIS-UF SCA-8 with MW < 3 kDa reveals its promising potential use as a food biopreservative.

(5) $$\begin{array}{c} {}*\text{Residue activity}=\frac{\text{inhibition zone of threated sample}-6}{\text{inhibition zone of unthreated control}-6}\times 100. \end{array}$$

### 4.2. Median Lethal Concentration of BLIS Ultrafiltration SCA-8 with MW < 3 kDa Fraction

The most promising BLIS fraction, BLIS-UF SCA-8 with MW < 3 kDa, was subjected to the brine shrimp lethality assay to determine its LC_50_ [41]. The test results showed that the mortality rate of *A. salina* increased with increasing BLIS concentration, in which a probit test showed that this fraction has LC_50_ of 226.975 *μ*g·mL^−1^ (moderate toxicity) (Table 5). Hamidi et al. [42] classified toxicity based on LC_50_ as follows: highly toxic, 0–100 *μ*g·mL^−1^; moderately toxic, 100–500 *μ*g·mL^−1^; weakly toxic, 500–1,000 *μ*g·mL^−1^; and nontoxic, >1,000 *μ*g·mL^−1^. The LC_50_ of BLIS-UF SCA-8 with MW < 3 kDa was higher than that of the purified bacteriocin from *Lactobacillus lactis*, which revealed an LC_50_ of 21.54 *μ*g·mL^−1^ [43]. The difference in LC_50_ between BLIS samples may be attributed to differences in the purity of the samples.

LC_50_ in *A. salina* and LD_50_ in animal models has a positive correlation, in which LC_50_ < 10 *μ*g · mL^−1^ has an LD_50_ of 100–1,000 mg·kg^−1^, LC_50_ < 20 *μ*g · mL^−1^ has an LD_50_ of 1,000–2,500 mg·kg^−1^, and LC_50_ > 25 *μ*g · mL^−1^ has an LD_50_ of 2,500–8,000 mg·kg^−1^ [44]. On the basis of these studies, BLIS-UF SCA-8 with MW < 3 kDa may be assumed to have an LD_50_ of over 2500 mg·kg^−1^ (weakly toxic) because its LC_50_ to *A. salina* is 226.975 *μ*g·mL^−1^. Erhirhie et al. [45] classified LD_50_ according to the dose range as follows: extremely toxic, <5 mg·kg^−1^; highly toxic, 5–50 mg·kg^−1^; moderately toxic, 50–500 mg·kg^−1^; weakly toxic, 500–5,000 mg·kg^−1^; nontoxic, 5000–15,000 mg·kg^−1^, and relatively harmless, >15,000 mg·kg^−1^. These findings demonstrate that BLIS-UF SCA-8 with MW < 3 kDa may be developed as a promising preservative agent.

### 4.3. Identification of the Peptide Sequence of the BLIS Ultrafiltration SCA-8 with MW < 3 kDa Fraction

**Figure 5:**
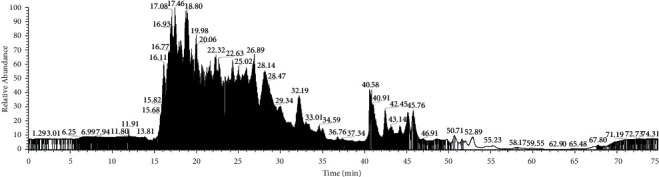
LC-MS/MS chromatogram profile of BLIS UF-SCA-8 with MW < 3 kDa fraction.

Peptide identification by LC-MS/MS can provide information on the molecular mass of the amino acid sequence of the peptide constituents of a sample [42]. LC-MS/MS of BLIS-UF SCA-8 with MW < 3 kDa followed by database-based protein analysis using Proteome Discoverer 2.2 succeeded in identifying 130 proteins (retention times of 15.21–19.98 min) (Figure 5; Table 6). Eleven peptides with over 60% relative abundance and MW ranging from 1.34 kDa to 2.90 kDa appeared to be responsible for the antibacterial activity of this fraction (Table 6). *In silico* analysis of physicochemical properties using ProtParam (https://web.expasy.org/protparam/) and PepDraw (http://www.tulane.edu/~biochem/WW/PepDraw/) revealed that each peptide having hydrophobic amino acids ranging from 38.5 to 75%,and high hydrophobicity (15.34–26.5 kcal·mol^−1^) (Table 6). Gadde et al. [46] found that antimicrobial peptides are generally hydrophobic and cationic. Cationic properties may help promote interactions between peptides and bacterial cell membranes or walls [47]. Hydrophobic properties could destroy cell walls or membranes by forming pores, resulting in cell lysis [48]. Protein-binding identification (Boman index) was conducted using an APD-based prediction program (http://aps.unmc.edu/AP/main.php). The detected proteins revealed high protein binding potential, especially those detected at 16.77 min (Table 6). Boman [49] stated that if a peptide or protein has a positive Boman index and is over 2.48 kcal·mol^−1^, the protein or peptide is highly likely to be able to bind to bacterial cell membranes.

Bacteriocins may be divided into three classes. Class I includes bacteriocins with posttranslational modified amino acid, which heat stable, have a low MW (<5 kDa), and are only able to inhibit Gram-positive bacteria. Class II includes bacteriocins with low MW (<10 kDa), heat stable, which do not contain a posttranslational modified amino acid and can inhibit both Gram-positive and negative bacteria. Finally, class III includes bacteriocins with large MW (>30 kDa) and is generally heat labile [50]. According to this classification and the data obtained in the present study, BLIS-UF SCA-8 with MW < 3 kDa may be a class II bacteriocin. This assumption is based on the MW of BLIS-UF SCA-8 (<3 kDa), its heat stable characteristics, its ability to inhibit Gram-positive and negative bacteria, and the absence of a posttranslational modified amino acid in its peptide sequence.

## 5. Conclusions

*Streptomyces* is a promising producer of bioactive metabolites. The present study showed that six isolates of *Streptomyces* isolated from the gut of *C. chanos* could produce BLIS. The proteinaceous properties of BLIS were confirmed with assays involving several proteolytic enzymes. Crude BLIS showed broad spectrum antibacterial activity and high temperature and pH stability. Fractionation with ultrafiltration membranes revealed that BLIS fractions with MWs of <3 kDa and 3–10 kDa present the greatest activity. Calculation of the specific activities of these fractions demonstrated that BLIS-UF SCA-8 with MW < 3 kDa was more active than the fraction with MW of 3–10 kDa. BLIS-UF SCA-8 with MW < 3 kDa showed an increase in purity of up to 2.5 times, good pH and heat stability, and moderate toxicity to *A. salina*. Identification of the peptide sequence of this fraction through LC-MS/MS revealed that the peptides of this fraction are mostly composed of hydrophobic amino acids with low MW. Overall, the results of this study demonstrate the promising potential of BLIS *Streptomyces* as a candidate antibacterial agent for food applications.

## Figures and Tables

**Table 1:** Inhibitory zone (mm) of crude BLIS after proteolytic enzyme treatment against *Escherichia coli.*

Treatment	Inhibition zone (mm)					
	SCA-5	SCA-8	SCA-11	AIA-10	AIA-12	AIA-17
Proteinase-K	0	0	0	0	0	0
Pepsin	0	7.0 ± 0.32	0	7.3 ± 0.03	6.4 ± 0.07	7.4 ± 0.10
Trypsin	0	0	0	0	0	0
Untreated control	19.2 ± 0.10	16.5 ± 0.13	24.1 ± 0.12	22.6 ± 0.05	21.7 ± 0.18	20.4 ± 0.19

**Table 2:** Total protein (*μ*g), total BLIS activity (AU), BLIS-specific activity (AU·*μ*g^−1^), purification level, and yield (%) during fractionation steps of BLIS produced by *Streptomyces* sp.

Isolates	Purification step		Volume (mL)	Total protein (*μ*g)	Total BLIS activity (AU)^a^				BLIS-specific activity (AU·*μ*g^−1^)^b^				Purification level^c^				Yield (%)^d^			
					EC	SA	LM	ST	EC	SA	LM	ST	EC	SA	LM	ST	EC	SA	LM	ST
SCA-5	CFS		1,200	15,029	ND	ND	ND	ND	ND	ND	ND	ND	ND	ND	ND	ND	ND	ND	ND	ND
	Crude BLIS		100	2,914	424,094	131,187	765,890	492,080	145.5	45.0	262.8	168.9	1.0	1.0	1.0	1.0	100	100	100	100
	BUF (kDa)	>10	80	2,741	264,296	131,152	616,528	453,048	96.4	47.8	224.9	165.3	0.7	1.1	0.9	1.0	62.3	100.0	80.5	92.1
		3–10	80	1,113	224,456	118,144	627,040	340,208	201.6	106.1	563.2	305.6	1.4	2.2	2.1	1.8	52.9	90.1	81.9	69.1
		<3	80	845	157,792	93,152	387,312	397,928	186.8	110.3	458.6	471.2	1.3	1.0	1.7	2.8	37.2	71.0	50.6	80.9
SCA-8	CFS		1,200	14,044	ND	ND	ND	ND	ND	ND	ND	ND	ND	ND	ND	ND	ND	ND	ND	ND
	Crude BLIS		100	2,305	850,141	322,553	616,900	861,330	368.8	139.9	267.6	373.6	1.0	1.0	1.0	1.0	100	100	100	100
	BUF (kDa)	>10	80	1,793	672,344	244,136	614,088	785,864	375.0	136.2	342.5	438.3	1.0	1.0	1.3	1.2	79.1	75.7	99.5	91.2
		3–10	80	858	469,152	242,688	567,784	533,010	546.7	266.2	622.9	584.7	1.5	1.9	2.3	1.6	55.2	75.2	92.0	61.9
		<3	80	912	579,334	144,112	570,245	528,174	635.6	167.9	664.5	615.5	1.7	1.2	2.5	1.6	68.1	44.7	92.4	61.3
SCA-11	CFS		1,200	21,790	ND	ND	ND	ND	ND	ND	ND	ND	ND	ND	ND	ND	ND	ND	ND	ND
	Crude BLIS		100	2,332	722,526	253,013	602,200	664,730	309.9	108.5	258.3	285.1	1.0	1.0	1.0	1.0	100	100	100	100
	BUF (kDa)	>10	80	2,287	664,184	239,928	598,320	661,792	290.4	104.9	261.6	289.4	0.9	1.0	1.0	1.0	91.9	94.8	99.4	99.6
		3–10	80	1,098	569,024	232,968	565,294	575,300	518.2	212.2	514.8	523.9	1.7	2.0	2.0	1.8	78.8	92.1	93.9	86.5
		<3	80	899	385,736	130,016	421,728	585,505	429.2	144.7	469.2	651.5	1.4	1.3	1.8	2.3	53.4	51.4	70.0	88.1
AIA-10	CFS		1,200	20,997	ND	ND	ND	ND	ND	ND	ND	ND	ND	ND	ND	ND	ND	ND	ND	ND
	Crude BLIS		100	2,237	631,981	137,861	414,090	308,081	282.6	61.6	185.2	137.8	1.0	1.0	1.0	1.0	100	100	100	100
	BUF (kDa)	>10	80	2,228	550,840	116,624	330,354	297,728	247.2	52.3	148.3	133.6	0.9	0.8	0.8	1.0	87.2	84.6	79.8	96.6
		3–10	80	1,288	429,639	95,344	226,938	185,438	333.5	74.0	176.2	144.0	1.2	1.2	1.0	1.0	68.0	69.2	54.8	60.2
		<3	80	1,059	264,728	33,768	64,914	305,260	250.0	31.9	61.3	288.3	0.9	0.5	0.3	2.1	41.9	24.5	15.7	99.1
AIA-12	CFS		1,200	19,219	ND	ND	ND	ND	ND	ND	ND	ND	ND	ND	ND	ND	ND	ND	ND	ND
	Crude BLIS		100	2,327	564,889	115,696	497,410	399,570	242.8	49.7	213.8	171.7	1.0	1.0	1.0	1.0	100	100	100	100
	BUF (kDa)	>10	80	2,141	538,424	93,624	432,776	397,912	251.5	43.7	202.2	185.9	1.0	0.9	0.9	1.1	95.3	80.9	87.0	99.6
		3–10	80	910	477,715	60,080	487,506	275,671	525.0	66.0	535.8	303.0	2.2	1.3	2.5	1.8	84.6	51.9	98.0	69.0
		<3	80	794	326,776	18,200	151,688	307,120	411.4	22.9	191.0	386.7	1.7	0.5	0.9	2.3	57.8	15.7	30.5	76.9
AIA-17	CFS		1,200	17,060	ND	ND	ND	ND	ND	ND	ND	ND	ND	ND	ND	ND	ND	ND	ND	ND
	Crude BLIS		100	2,250	495,133	269,670	355,920	312,520	220.1	119.9	158.2	138.9	1.0	1.0	1.0	1.0	100	100	100	100
	BUF (kDa)	>10	80	1,946	214,480	145,008	254,319	288,400	110.2	74.5	130.7	148.2	0.5	0.6	0.8	1.1	43.3	53.8	71.5	92.3
		3–10	80	1,332	388,558	129,688	193,157	305,264	291.8	97.4	145.0	229.2	1.3	0.8	0.9	1.7	78.5	48.1	54.3	97.7
		<3	80	833	295,272	98,000	21,457	271,976	354.5	117.6	25.8	326.5	1.6	1.0	0.2	2.4	59.6	36.3	6.0	87.0

^∗^CFS: cell-free supernatant; BUF: BLIS ultrafiltration fraction; ND: not determined; tested bacteria: *E. coli* (EC), *S. aureus* (SA), *L. monocytogenes* (LM), and *S.* Typhimurium (ST). ^a^Total BLIS activity was determined by agar well diffusion, considering the total sample volume. ^b^BLIS-specific activity was determined by the ratio between total BLIS activity and total protein content. ^c^Purification level was determined by the ratio between specific activity in each step and the specific activity of crude BLIS. ^d^Yield was determined by the ratio between total BLIS activity in each step and the total activity in crude BLIS.

**Table 3:** The MIC and MBC of BLIS-UF SCA-8 with molecular weight less than 3 kDa.

Bacterial test	MIC (mg·mL^−1^)	MBC (mg·mL^−1^)
*Escherichia coli* ATCC 25922	0.31	0.62
*Salmonella* Typhimurium ATCC 14028	0.62	1.25
*Staphylococcus aureus* ATCC 25923	0.04	0.08
*Listeria monocytogenes* ATCC 35152	0.04	0.16

**Table 4:** SCA-8 with MW < 3 kDa against *Escherichia coli.*

Stability test	Inhibition zone (mm)	Residue activity (%)^∗^
*Heat*		
60°C	23.78 ± 0.25	97.13
80°C	23.50 ± 0.07	95.63
100°C	18.90 ± 0.49	70.49
121°C	12.23 ± 0.32	34.02
*pH*		
2	22.80 ± 0.21	91.80
4	22.00 ± 0.28	87.43
6	23.53 ± 0.11	95.77
7	21.85 ± 0.42	86.61
8	14.83 ± 0.25	48.22
10	0	0
*Enzyme*		
Proteinase-K	0	0
Pepsin	6.35 ± 0.14	1.93
Trypsin	0	0
*Untreated control*	24.3 ± 0.14	100

**Table 5:** LC_50_ by the brine shrimp lethality test of most potential BLIS SCA-8 with MW < 3 kDa fraction.

Sample	Concentration (*μ*g·mL^−1^)	Mortality (%)	LC_50_ (*μ*g·mL^−1^)
BLIS SCA-8 with MW < 3	1000	96.6	226.975
	500	66.6	
	100	40	
	10	13.3	

**Table 6:** Identified peptide sequences (relative abundance > 60%) by Proteome Discoverer 2.2 (Thermo Scientific) in BLIS-UF SCA-8 with MW < 3 kDa fraction.

RT (min)	Relative abundance (%)	Accession	Sequence	Molecular weight (Da)	Number of amino acid	Hydrophobicity (kcal·mol^−1^)	Boman index
16.11	61.67	F2IY40	TAVTEWTVAHAGGER	1584.77	15	20.84	1.56
16.52	61.37	A0A2A4MKN2	VTMNKEIDGLER	1420.71	12	22.16	1.16
16.77	69.94	A0A087LGR3	EQLSAFLQSEEGK	1465.71	13	21.49	2.9
16.91	82	A0A1C9VDC5	SAGPMERIFASGMR	1345.66	14	15.34	2.17
16.93	86.02	K2NT65	AGGAYVPIDPGYPSER	1493.69	16	19.31	1.81
17.08	98.53	A0A087LFK0	EVGASSKADMGK	1195.56	12	23.86	1.92
17.46	100	A0A2G7E6K9	TAAGLEAGETVR	1174.60	12	19.56	1.17
18.07	77.3	A0A217EF20	QAAPYPLPADPDAR	1481.73	14	18.36	1.795
18.8	98.53	A0A1U9ZU28	AFDPPPTTHPPATDAR	1690.81	16	20.56	1.45
19.44	71.08	A0A397RL80	DLGAWPALPGEDTSTSSSGPTAAPSISAHQ	2908.35	30	26.22	1.84
19.98	80.64	A0A1B1AP88	RDAPGASPGGTAGRGGPR	1636.82	18	26.5	2.27

Accession: the master protein accession as found in the database (UniProt).

## Data Availability

All datasets generated or analyzed during this study are available upon reasonable request from the corresponding author.
